# Experiments and Observations on the Absorption of Chyle

**Published:** 1846-05

**Authors:** 


*Experiments and Observations on the Absorption of Chyle.*—M. Weber's labours go to show first, that absorption of chyle begins to be effected in the conical or cylindrical cellules of the epithelium of the intestinal mucous membrane; at a particular period of digestion those parts become swollen and filled with globules of chyle; the chyle proceeds by ducts still undiscovered into cellules placed beneath, and which envelope the lymphatics and bloodvessels; the lacteals finally take up the chyle in a manner equally unknown [endosmose?] 2d. The presence of chyle can be very satisfactorily traced in the rabbit and in the frog—in the former the chyle is white, in the latter yellow. 3d. Two large cells, composed of smaller cells and covered with epithelium, are often found in the human subject during digestion; one of those cells contain a white and opaque liquid, and the other a thick, transparent one; these two cells are in contact. [This observation has been made by others.] 4th. Most of the intestinal villi in the human subject enclose but one lymphatic vessel, which goes to its free extremity; several of these vessels often go to the larger villi, anastomosing freely with each other in their course; if any of the large villi are divided at their extremity into two, the lymphatic vessel also divides and sends a branch to each portion of the villus. 5th. Very small, chyliferous vessels situated in the villus take their origin in the lymphatico-chyliferous vessels to be described; their diameter is equal to the capillary vessels of the villus; the network they form is compact—that is, there is no interstice between the vessels which compose them. 6th. When the network of the smaller lymphatic vessels are dilated and filled with chyle, they render the villi opaque; one may explain by this fact how many anatomists have taken the central chyliferous vessel and the terminal network as one ampulla filled with chyle. 7th. There are in those already discernible chyle containing masses of thick globules perfectly analogous both in size and colour to the chyle contained in the lymphatic vessels.—*Ibid.*

